# Reported number of sexual partners: comparison of data from four African longitudinal studies

**DOI:** 10.1136/sti.2008.033985

**Published:** 2009-03-13

**Authors:** J Todd, I Cremin, N McGrath, J-B Bwanika, A Wringe, M Marston, I Kasamba, P Mushati, T Lutalo, V Hosegood, B Żaba

**Affiliations:** 1 Medical Research Council/Uganda Virus Research Institute, Uganda Research Unit on AIDS, Entebbe, Uganda; 2 Imperial College London, UK; 3 London School of Hygiene and Tropical Medicine, London, UK; 4 Africa Centre for Health and Population Studies, University of KwaZulu Natal, South Africa; 5 Rakai Health Science Program, Entebbe, Uganda; 6 Biomedical Research and Training Institute, Harare, Zimbabwe

## Abstract

**Objective:**

To compare reported numbers of sexual partners in Eastern and Southern Africa.

**Methods:**

Sexual partnership data from four longitudinal population-based surveys (1998–2007) in Zimbabwe, Uganda and South Africa were aggregated and overall proportions reporting more than one lifetime sexual partner calculated. A lexis-style table was used to illustrate the average lifetime sexual partners by site, sex, age group and birth cohort. The male-to-female ratio of mean number of partnerships in the last 12 months was calculated by site and survey. For each single year of age, the proportion sexually active in the past year, the mean number of partners in the past year and the proportion with more than one partner in the past year were calculated.

**Results:**

Over 90% of men and women between 25 and 45 years of age reported being sexually active during the past 12 months, with most reporting at least one sexual partner. Overall, men reported higher numbers of lifetime sexual partners and partners in the last year than women. The male-to-female ratio of mean partnerships in the last year ranged from 1.41 to 1.86. In southern African cohorts, individuals in later birth cohorts reported fewer sexual partners and a lower proportion reported multiple partnerships compared with earlier birth cohorts, whereas these behavioural changes were not observed in the Ugandan cohorts. Across the four sites, reports of sexual partnerships followed a similar pattern for each sex.

**Conclusions:**

The longitudinal results show that reductions in the number of partnerships were more evident in southern Africa than in Uganda.

The HIV/AIDS epidemic has prompted an increase in sexual behaviour surveys. Understanding the relationship between sexual behaviour and the transmission of HIV and other sexually transmitted infections (STIs) in sub-Saharan Africa is essential to better understanding the dynamics of the HIV epidemic.^1^ At an individual level, within a defined population, the overall number and rate of acquisition of sexual partners are associated with an increased risk of HIV and other STIs.^2^ However, at a population level, the aggregated number of lifetime sexual partners is broadly similar across different countries.^3^ Furthermore, an ecological cross-sectional study of four African cities showed no association between the mean number of lifetime sexual partners and the prevalence of HIV infection in the population.^4^

However, sexual behaviour is known to change over time and has been associated with declines in the prevalence of HIV in a number of countries.^5^^–^7 A comparison of longitudinal studies in different populations would be useful to see if behaviour change is occurring and if the patterns of reported sexual behaviour change are similar in different settings.

This paper compares reported sexual partnerships over a 10-year period across four sites in Africa, examining differentials by age, gender and site. Using two common definitions—(1) reported number of lifetime sexual partners and (2) reported number of sexual partners in the past 12 months—we can compare patterns of sexual behaviour in these populations across time. Since HIV is transmitted mainly through heterosexual sex in these populations, any differences identified can be used to enhance our understanding of the heterogeneity of the HIV epidemic.

## METHODS

### Data sources

Four HIV cohort studies participating in the ALPHA network^8^ contributed data from adults (aged 15 years and older) for this analysis. All sites collected data on number of lifetime sexual partners in at least one survey, and data on sexual partners in the past 12 months in several surveys. Different sites asked the questions in a different way and used different selection criteria. However, within each site, data were used where similar questions had been asked in a consistent order across different survey rounds. Questionnaires and protocols are available through the ALPHA network.^8^

In the Masaka cohort in south-western Uganda, an open cohort was started in 1989 and annual rounds of data collection have been maintained with data to 2005 available for this analysis.^9^ ^10^ Sexual behaviour data including lifetime sexual partners were collected in 2000–1 and 2006–7. Data on the number of sexual partners in the past 12 months were collected in 10 of the 11 annual survey rounds between 1996–7 and 2006–7, with a consistent order within the structured questionnaire.

In the Manicaland cohort in eastern Zimbabwe, three survey rounds were completed between 1998/2000, 2001/3 and 2003/5.^6^ ^11^ Data from all three surveys are used in this analysis. In the first survey, sexual behaviour data were collected using a face-to-face interview for 22.6% of men and 26.9% of women, and the remainder were interviewed using an informal confidential voting method.^12^ The proportion of respondents interviewed using the informal confidential voting method increased over time as all new literate respondents were interviewed using this method. In all rounds, data on both lifetime sexual partners and sexual partners in the last year were collected using the same data collection methods.

In the Africa Centre, demographic surveillance is conducted in a circumscribed population within Umkhanyakude district in KwaZulu Natal, South Africa.^13^ Four behavioural survey rounds were completed between 2003 and 2007,^14^ collecting data on number of sexual partners in the past 12 months in each round and lifetime sexual partner data only in the first round (2003–4). In the first round a secret voting method similar to that used in Manicaland was offered to participants.^12^

The Rakai Health Sciences Program (RHSP) in south-western Uganda has conducted annual surveys using an open community cohort of consenting persons aged 15–49 years since 1994.^15^ ^16^ Surveys have been conducted annually using a questionnaire that includes modules on both sociodemographic and behavioural characteristics. Questions on lifetime sexual partners and sexual partners in the past 12 months have been asked using the same protocol between rounds 6 (1999/2000) and 11 (2005/6).^15^

### Data analysis

Age at survey was recorded in each study by self-reported age, date of birth, or both. For each study, data from each survey were combined and analysed for each sex separately. For these analyses, all study participants were used including those who had never had sex (virgins) and those abstaining from sex for any reason including postpartum abstinence. Trends over time within sites are described, but no formal statistical comparisons have been made between sites. The reported number of lifetime sexual partners was truncated at 30 to reduce the impact of outliers on the estimates of mean number of lifetime partners.

The proportion of respondents reporting more than one lifetime sexual partner was analysed using 10-year age groups, starting with respondents aged 15–24 years and combining all respondents over 55 years of age. The average number of lifetime partners was illustrated using a lexis-style table, with 10-year birth cohorts, showing the responses in 5-year age groups. For this table, the earliest birth cohort consisted of participants born before 1950 for Masaka and Manicaland and participants born between 1950 and 1959 for Umkhanyakude and Rakai. The average number of lifetime partners was inversely weighted by the number of times respondents contributed reports in the same age group.

For each site the total number of partnerships and the mean number of partners reported in the past 12 months were calculated for all participants, excluding virgins, by sex and survey round. For each survey round the mean number of partnerships in the past 12 months was used to calculate a male-to-female reporting ratio.

The analyses of partners in the past 12 months used sex-specific 5-year birth cohorts, with the earliest cohort consisting of all participants born before 1960. Reports from all surveys were aggregated and, for each age (in single years), the mean number of partners in the last year, the proportion who were sexually active in the past 12 months and the proportion with more than one partner in the last year were calculated and the results presented graphically.

## RESULTS

### Lifetime sexual partners

In all sites and in all surveys, close to 100% of respondents aged >25 years reported at least one lifetime sexual partner. More than one lifetime partner was reported by >72% of male respondents aged 25 years or older in Manicaland and Umkhanyakude, but lower proportions were observed in Masaka, peaking at 56% in 2001 and 69% in 2006 among men aged 25–34 years (table 1). Among men aged 25 years or older in Rakai, >90% reported more than one lifetime sexual partner, with little change over the six surveys. Among women aged 25 years or more, the proportion reporting more than one lifetime partner was lower than for men, with around 30% in Manicaland, 40% in Masaka, 50% in Umkhanyakude and 70% in Rakai (table 1). Less than 2% of male respondents and less than 0.5% of female respondents in each site reported more than 30 lifetime sexual partners.

**Table 1:** Proportion of respondents reporting more than one lifetime sexual partner by site, survey, sex and 10-year age group

Age group	Masaka		Manicaland			Umkhanyakude	Rakai					
	1998–1999	2005–2006	1998–2000	2001–2003	2003–2005	2003–2004	1999–2000	2000–2001	2001–2002	2002–2003	2003–2004	2005–2006
*Men*												
15–24	25.3	13.6	77.0	34.6	31.6	29.0	65.1	67.4	68.6	65.7	65.9	58.5
25–34	56.0	69.4	87.0	72.4	83.2	83.0	93.4	94.4	94.2	92.6	93.2	94.3
35–44	54.4	67.2	90.0	90.6	90.8	90.0	96.4	97.1	97.7	95.4	96.5	96.5
45–54	47.4	67.9	89.4	89.8	90.3	91.0	98.5	97.2	98.2	96.8	97.7	97.9
55+	38.4	52.6	–	–	–	–	98.7	98.1	99.2	–	–	–
N	1595	2448	4281	3362	6491	6245	6603	6346	7213	5113	4804	5654
												
*Women*												
15–24	14.2	8.6	25.7	10.4	12.6	16.0	52.4	52.4	56.1	52.7	54.6	54.3
25–34	39.9	30.6	33.9	30.4	33.8	56.0	75.5	74.4	77.2	75.6	74.9	75.9
35–44	42.1	42.6	24.0	24.8	32.4	59.0	79.3	80.0	80.7	80.9	80.0	82.9
45–54	31.0	41.6	18.4	20.7	23.0	55.0	77.3	79.1	82.1	79.8	82.1	82.5
55+	14.0	25.7	–	–	–	–	67.9	60.9	66.7	–	–	–
N	1976	3313	5249	4848	9775	11922	8489	8263	9073	6929	7076	8103

Age ranges: Masaka: males and females 13 years and over. Manicaland: males 17–54 years, females 15–44 years, extended to 15–54 years for males and females in the third survey. Umkhanyakude: males 15–54 years and females 15–49 years. Rakai: males and females 15 years and over.

In all sites and in all birth cohorts, men aged >35 years reported an average of >6 lifetime sexual partners (table 2). Among women born in the 1960s or earlier, the mean reported number of lifetime partners was lower in the Southern African sites (range 1.3–2.2) compared with the East African sites (range 1.7–3.6). However, in all sites, men in the later birth cohorts had fewer lifetime sexual partners than earlier birth cohorts within each age group, although this reduction in reported lifetime sexual partners was not seen in later birth cohorts of women. The magnitude of this effect was large in the younger men in all four sites. Among men born in the 1950s and 1960s there was some reduction in the reported number of lifetime sexual partners over time, but these trends were not significant.

**Table 2:** Reported number of lifetime sexual partners (mean and number reporting) for 10-year birth cohorts by each 5-year age group and for each sex separately

	Men								Women							
	15–19	20–24	25–29	30–34	35–39	40–44	45–49	50–54	15–19	20–24	25–29	30–34	35–39	40–44	45–49	50–54
*Masaka*																
Before 1950								10.2							1.7	2.2
								36							3	77
1950–9						10.1	9.3	7.9					2.0	3.0	2.5	2.7
						65	98	109					3	95	209	141
1960–9			6.0	9.0	8.5	7.4	7.8				1.0	2.9	2.9	2.8	2.8	
			2	106	164	142	47				1	162	275	251	89	
1970–9	1.0	3.9	6.9	5.9	7.2				1.2	2.2	2.3	2.3	2.3			
	4	247	263	190	64				10	276	417	340	101			
1980–9	0.8	2.7	5.1						0.7	1.8	2.2					
	809	232	75						804	384	141					
*Manicaland*																
Before 1950							8.5	8.8							1.7	1.5
							22	173							18	101
1950–9					9.8	8.8	8.3	7.7					1.8	2.0	1.8	1.6
					25	291	510	252					60	639	1075	531
1960–9			6.8	7.8	7.9	7.2	11.6				1.9	2.2	2.1	2.0	1.3	
			72	520	835	423	5				99	749	1524	923	6	
1970–9	4.3	5.7	5.9	6.2	13.0				1.7	1.8	1.9	2.2	1.6			
	124	1192	1680	713	5				93	1125	2197	1094	18			
1980–9	1.1	3.2	3.5						0.5	1.4	1.7					
	2831	1400	8						3143	1806	21					
*Umkhanyakude*																
Before 1950																
1950–9						10.1	9.2	8.7						2.1	2.0	2.0
						50	266	146						262	1525	24
1960–9				5.7	6.5	7.6	9.1					1.9	2.1	2.1	1.9	
				74	308	259	12					254	1633	1760	296	
1970–9		4.2	6.1	6.5	6.1					1.8	2.0	2.0	1.9			
		122	555	385	28					298	1879	1705	270			
1980–9	0.9	2.6	4.7						0.3	1.0	1.6					
	3647	2025	92						5511	3654	302					
*Rakai*																
Before 1950																
1950–9							9.3	10.7							3.6	2.8
							137	18							340	54
1960–9					8.2	9.4	8.4						3.1	3.2	3.3	
					353	270	28						548	556	92	
1970–9			6.5	7.1	8.2						2.5	2.7	3.0			
			619	575	64						1176	1107	113			
1980–9	3.5	5.5	6.1						1.8	2.3	2.1					
	207	545	121						307	1061	206					

### Sexual partners in the past 12 months

The mean number of reported sexual partners in the past 12 months for non-virgin men across the different sites and between the different rounds ranged from 1.19 (in Umkhanyakude) to 1.90 (in Manicaland) (table 3). For non-virgin women, the overall average number of reported partners was lower than for males and varied between 0.82 (in Umkhanyakude) and 1.09 (in Rakai). The decline over time in the mean number of partnerships in Umkhanyakude may be partially attributable to the change in method used to collect behavioural data. The male-to-female ratio for the mean number of partnerships was reasonably consistent at around 1.60 (range 1.41–1.86) for all sites, with an overall average of 1.50 indicating that, on average, men report 50% more partners than women.

**Table 3:** Total partnerships reported in past 12 months by non-virgin men and women in each survey in each site

Site	Survey year	Partnerships reported by non-virgin particpants		Male/female reporting ratio
		By men	By women	Ratio of means
		Total (mean)	Total (mean)	
Masaka				
	1996/7	1395 (1.55)	993 (1.03)	1.50
	1998/9	1939 (1.66)	1290 (1.06)	1.57
	1999/00	2785 (1.68)	2034 (1.05)	1.60
	2000/1	2979 (1.68)	2150 (1.04)	1.62
	2001/2	2704 (1.62)	2194 (1.04)	1.56
	2002/3	2441 (1.54)	2063 (1.04)	1.48
	2003/4	2422 (1.51)	2154 (1.05)	1.44
	2004/5	2147 (1.52)	1928 (1.03)	1.48
	2005/6	2177 (1.57)	1938 (1.03)	1.52
Manicaland				
	1998/00	6582 (1.90)	4222 (1.02)	1.86
	2001/3	3695 (1.38)	3477 (0.98)	1.41
	2003/5	6758 (1.46)	7088 (0.92)	1.59
Umkhanyakude				
	2003/4	8031 (1.56)	13870 (0.85)	1.84
	2005	6136 (1.26)	14460 (0.85)	1.48
	2006	5598 (1.23)	13335 (0.83)	1.48
	2007	3970 (1.19)	10836 (0.82)	1.45
Rakai				
	1999/00	9060 (1.76)	7120 (1.08)	1.63
	2000/1	9742 (1.80)	7372 (1.07)	1.68
	2001/2	11394 (1.83)	8289 (1.09)	1.68
	2002/3	7643 (1.78)	6128 (1.06)	1.68
	2003/4	7096 (1.74)	6268 (1.06)	1.64
	2005/6	8089 (1.74)	7013 (1.07)	1.63

**Figure 1:**
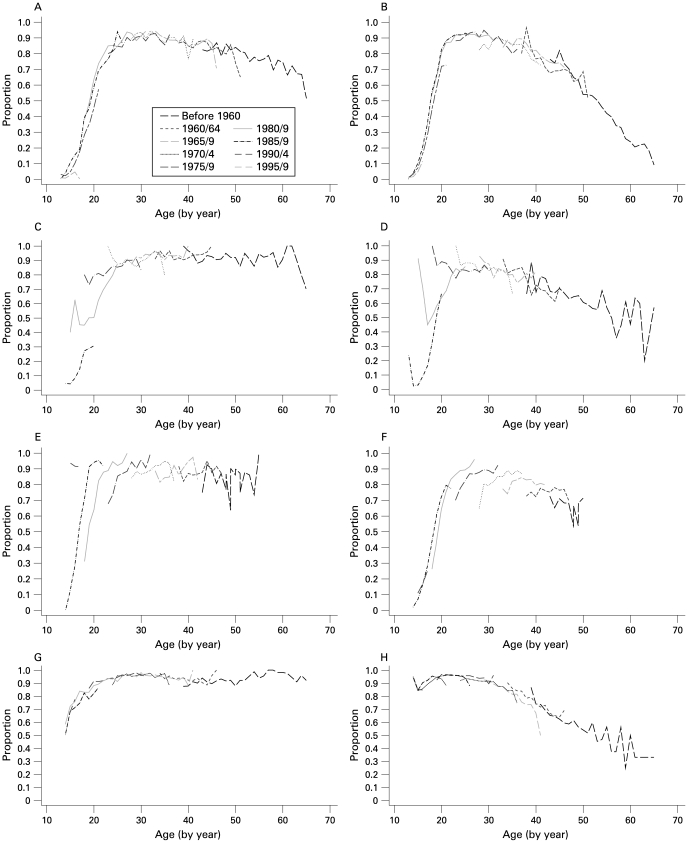
Proportion of respondents who reported being sexually active in the past 12 months by age and 5-year birth cohort. (A) Masaka men; (B) Masaka women; (C) Manicaland men; (D) Manicaland women; (E) Umkhanyakude men; (F) Umkhanyakude women; (G) Rakai men; (H) Rakai women.

By the age of 25, over 90% of men reported being sexually active in the past year in all sites, with this level attained slightly earlier in Umkhanyakude (fig 1). In Umkhanyakude, Manicaland and Rakai, about 90% of men aged ⩾50 years reported sexual activity during the past year, whereas in Masaka this proportion decreased with age with fewer than 75% reporting being sexually active after the age of 50 years. For women in the four sites, 90% reported being sexually active in the past year by the age of 20 years, with this proportion being reached at an earlier age in Rakai. The proportion of women who reported being sexually active in the past year declined in all sites after the age of 40. By the age of 50 years, <50% of women reported being sexually active in Masaka and Rakai. In Manicaland and Umkhanyakude a similar but smaller decline in the proportion who reported being sexually active at older ages was observed.

**Figure 2:**
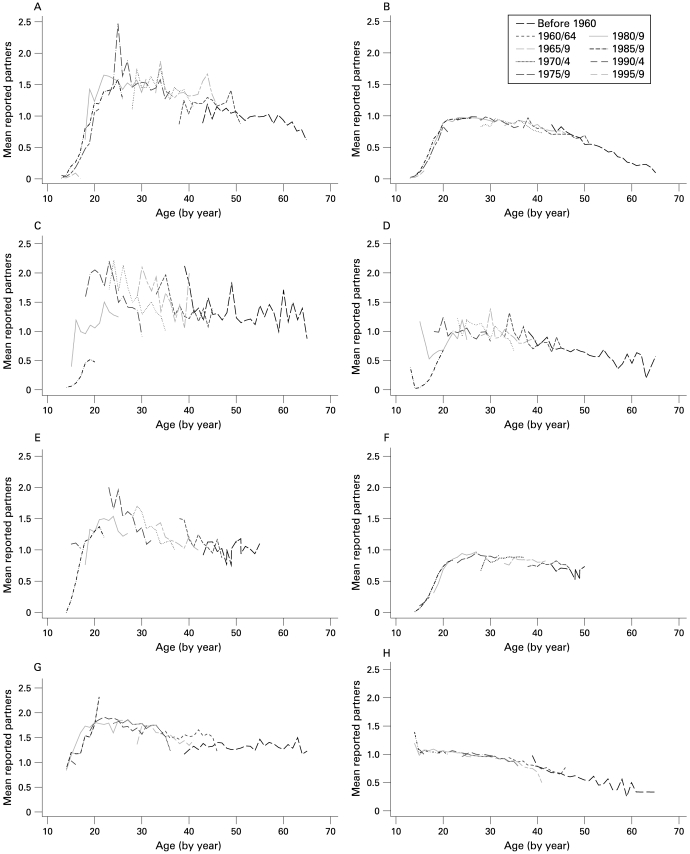
Mean number of reported sexual partners in past 12 months by birth cohort and age. (A) Masaka men; (B) Masaka women; (C) Manicaland men; (D) Manicaland women; (E) Umkhanyakude men; (F) Umkhanyakude women; (G) Rakai men; (H) Rakai women.

The mean number of sexual partners in the past 12 months for each site is shown in fig 2. In Masaka and Rakai there was remarkable consistency between the different birth cohorts for both sexes. In all sites, men averaged around 1.5 sexual partners per year at 25 years of age, decreasing to one per year at the age of 50 in Masaka and Umkhanyakude and a slightly smaller decrease in Rakai and Manicaland. In Manicaland and Umkhanyakude, men in later birth cohorts reported fewer partners in the past 12 months at each age than those in earlier birth cohorts, with some suggestion of a decrease in later birth cohorts in Masaka. In all sites, women reported fewer partners in the past 12 months than men, with about one per year at 25 years of age, decreasing to 0.5–0.8 per year by the age of 50 in all birth cohorts, although the decrease was less apparent in Rakai (fig 2).

**Figure 3:**
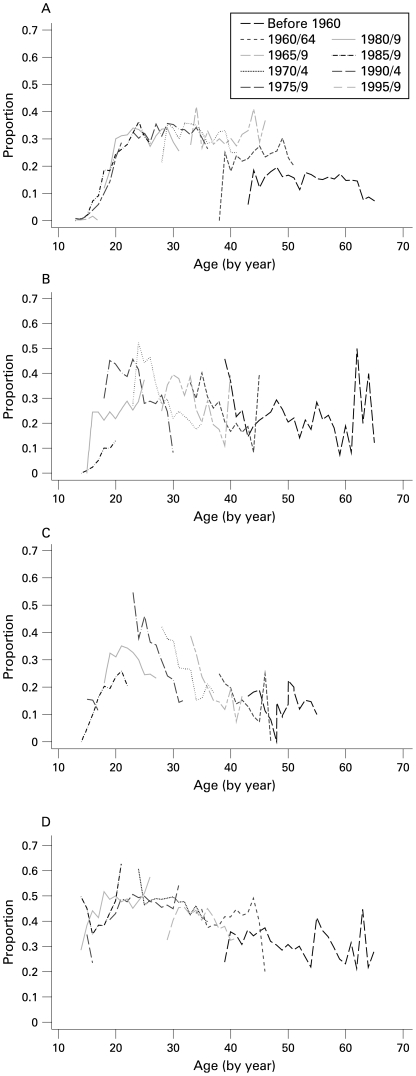
Proportion of men reporting more than one sexual partner in the past 12 months. (A) Masaka men; (B) Manicaland men; (C) Umkhanyakude men; (D) Rakai men.

In all age groups and in all birth cohorts, <5% of women reported more than one sexual partner in the past 12 months. In Masaka around 30% of men in all but the earliest birth cohorts reported more than one sexual partner in the past year (fig 3). In Manicaland the proportion of men reporting more than one partner in the last year declined over time from 40% to 20% across birth cohorts born in 1970–4 and 1975–9, with smaller declines observed in earlier birth cohorts. In Umkhanyakude, similar declines were observed among men in all age groups across birth cohorts. A higher proportion of men in Rakai reported more than one sexual partner in the past 12 months compared with the other three sites, with 50% at the age of 25 years decreasing to 30% at older ages.

## DISCUSSION

In this paper we have focused on two commonly used behavioural indicators—the number of lifetime sexual partners and the number of sexual partners in the last 12 months—and compared responses from four studies in Eastern and Southern Africa. Data have been provided from responses to questions asked at least once within four large population-based cohort studies that were set up to answer questions about HIV prevalence, incidence and longitudinal changes in sexual behaviour. Previous papers from these sites have described the studies and documented changes in sexual behaviour.^6^ ^7^ ^17^^–^19

The number of reported lifetime partners showed several similarities across age groups in the four sites. In all sites, at older ages (>35 years), men reported, on average, around 10 lifetime sexual partners, which is consistent with findings from DHS surveys in Uganda (10.6 lifetime sexual partners for men aged 40–49 years in 2004) and Zimbabwe (8.0 lifetime sexual partners for men aged 40–49 years in 2005).^20^ ^21^ Women reported a much lower average of 2 or 3 lifetime partners in all sites, which again is similar to the findings reported by Wellings *et al*.^3^

Comparing birth cohorts of the same age, among men, those in later birth cohorts reported fewer lifetime sexual partners than those in earlier birth cohorts. However, among women there was less evidence of a decrease in the later birth cohorts compared with the earlier birth cohorts. Within some male birth cohorts, particularly those born in Masaka in the 1950s and 1960s, a decline in the mean reported number of lifetime sexual partners was observed with increasing age. This may be due to misreporting of lifetime partnerships as 25% of both men and women in Masaka reported fewer lifetime partners in a later survey compared with the earlier survey. However, selective mortality may also contribute to this decline, whereby those individuals with high numbers of partners are removed from the population via AIDS mortality. Furthermore, all sites used open cohort members in the analysis rather than restricting to a closed cohort, and thus changing participation may also contribute to this observation.

For both men and women there are some small differences in the proportion reporting more than one lifetime sexual partner between the sites. In Masaka, fewer men at all ages reported more than one lifetime sexual partner compared with other sites. In Rakai, more women across all age groups reported having more than one lifetime partner. In Umkhanyakude, a higher proportion of women aged >24 years reported more than one lifetime partner, which may be related to the low rates of marriage and later age at first marriage in this population.^19^ ^22^ Despite these differences between the sites in reported lifetime partners, there is a consistency in the proportion of men who reported more than one sexual partner in the past 12 months. This suggests that reported partnerships in the past 12 months may be a more reliable measure than reported lifetime sexual partnerships, as these reports are less likely to be influenced by recall bias.

The median age at first sex for women is about 5 years younger than for men in Uganda and Umkhanyakude.^23^ ^24^ However, by the age of 25 years, over 90% of both men and women reported having a sexual partner during the past 12 months and can be said to be sexually active. In older women (>40 years), a decline in the proportion that is sexually active was observed in all sites. However, in older men, such a decline in the proportion that is sexually active was only seen in the Masaka site in Uganda and was not seen in most countries included in a large comparative analysis by Wellings *et al*.^3^ In the Manicaland and Umkhanyakude cohorts there was some evidence of a reduction in the reported number of sexual partners in the past 12 months in later birth cohorts, especially among the younger age groups. Others have reported declines in the number of sexual partners in Uganda in the mid 1990s, which is before the period considered in this paper.^17^ ^18^

In all four sites there was a striking gender differential with respect to reported number of partners and the proportion reporting more than one sexual partner, for both lifetime partners and partners in the past 12 months. Wellings *et al* point out that this could naturally arise through the population age structure in Africa and the patterns of age mixing, whereby older men have sex with younger women.^3^ However, Boerma *et al* note that community cohort surveys may miss highly sexually active women and sexually inactive men because of high levels of migration and mobility among these groups,^25^ or because of selection biases in sexual behaviour surveys. Many recent sexual behaviour surveys in Africa are conducted as part of HIV surveillance, and individuals with higher numbers of sexual partners may perceive themselves to be at higher risk of HIV and may be less likely to participate in the survey. In addition, women may under-report their number of sexual partners and men may tend to over-report.^26^ However, the proportion who are sexually active in the past year was similar for men and women in all four sites, and this did not change over time.

In all these sites there have been a number of HIV prevention interventions aimed at encouraging a reduction in the number of sexual partners. These interventions may have a different effect on sexual behaviour in men than in women. Results from Uganda showed that later birth cohorts of women (born in the 1980s) delayed sexual debut compared with earlier cohorts of women (born in the 1950s and 1960s), although this effect was not observed among men.^23^ In this paper we show that later cohorts of men have reported fewer sexual partners than earlier cohorts of men at the same age. Among men, the reduction in the number of reported sexual partners may have translated into lower demand for new partnerships with younger women, leading to less pressure on young women to start sex early and consequently later sexual debut for these women. More research on sexual mixing patterns in these sites is needed to explore this hypothesis.^27^ ^28^ Analysis of longitudinal data in this way illustrates trends in behaviour change. Further analyses and modelling are required to relate these changes in sexual behaviour to changes in the HIV prevalence in these populations.^6^

**Take-home messages**

- The patterns of reported sexual partners were consistent with each site, but differed by sex and by site.
- Across different surveys in all four sites, men reported 41–86% more sexual partners in the past year than women.
- For men, later birth cohorts reported fewer lifetime sexual partners than earlier birth cohorts at the same age in southern Africa, but not in Uganda.
- Taken by itself, the reported number of sexual partners is insufficient to explain the different levels of HIV infection.

There is much evidence linking reported number of sexual partners to the risk of HIV infection at an individual level.^29^^–^31 However, there is little evidence linking the number of sexual partners at a population level with the HIV prevalence in those populations.^27^ It has long been understood that African men do not have more sexual partners than men elsewhere.^3^ An ecological analysis of four cities with different levels of HIV prevalence showed that high-risk sexual behaviour, including a greater number of sexual partners, did not differ between the four cities.^32^ The limitation of the reported number of partners as a useful indicator of sexual behaviour has led researchers to analyse the types of partnership and levels of concurrency rather than the quantity of reported sexual partners.^28^ ^33^

The collection of sexual behaviour and partnership data is subject to several possible biases.^34^ Social desirability may encourage men to exaggerate their number of sexual partners while married women may remain secretive about their own additional partners,^26^ although this may be less applicable to South Africa where a lower proportion of women are married.^24^ An individual’s sexual behaviour varies over time, and different individuals may have periods of greater sexual activity and periods of abstinence, including postpartum abstinence. Thus, averaging the reported number of partners within a population will not accurately capture the changes that occur in individual sexual behaviour. This bias may change over time if losses to follow-up are higher among those with high numbers of sexual partners due to death or migration.^25^

The observed patterns over time demonstrate similarities across sites, and the longitudinal cohort design enables trends to be observed over time within each of the sites. Other explanatory data, such as marital status and migration patterns, that are not available for this comparative paper, can be used to explore further the patterns in behaviour within each of the sites and to explain how the behaviour is associated with the prevalence of HIV. At the population level, the reported number of sexual partners is remarkably similar across these different sites despite different levels of HIV infection. This would indicate that analysing the number of sexual partners is insufficient to explain the differences in the HIV epidemic in these sites, but more detailed analysis of the type of partners and the duration of partnerships is needed. Qualitative research results may also be useful in the interpretation of these data.

HIV prevention messages encourage abstinence and faithfulness within marriage. These data show some evidence that men have reduced the number of sexual partners in recent years. The large difference between the number of sexual partners (both lifetime and in the past 12 months) reported by men and women needs further research to identify the reasons for this apparent discrepancy.
